# Weighted Brain Network Analysis on Different Stages of Clinical Cognitive Decline

**DOI:** 10.3390/bioengineering9020062

**Published:** 2022-02-04

**Authors:** Majd Abazid, Nesma Houmani, Bernadette Dorizzi, Jerome Boudy, Jean Mariani, Kiyoka Kinugawa

**Affiliations:** 1 SAMOVAR, Télécom SudParis, Institut Polytechnique de Paris, 9 Rue Charles Fourier, F-91011 Evry, France; majd.abazid@telecom-sudparis.eu (M.A.); bernadette.dorizzi@telecom-sudparis.eu (B.D.); jerome.boudy@telecom-sudparis.eu (J.B.); 2 UMR 8256 Biological Adaptation and Aging, CNRS, Faculty of Sciences, Sorbonne University, F-75005 Paris, France; jean.mariani@sorbonne-universite.fr (J.M.); kiyoka.kinugawa@aphp.fr (K.K.); 3 Faculty of Medicine, Sorbonne University, F-75013 Paris, France; 4 Assistance Publique—Hôpitaux de Paris (AP-HP), DHU FAST, Functional Explorations and Sleep Investigation Unit for the Older Patients, Charles Foix Hospital, F-94200 Ivry-sur-Seine, France

**Keywords:** EEG signal, Alzheimer’s disease, subjective cognitive impairment, mild cognitive impairment, epoch-based entropy, graph theory, topological parameters

## Abstract

This study addresses brain network analysis over different clinical severity stages of cognitive dysfunction using electroencephalography (EEG). We exploit EEG data of subjective cognitive impairment (SCI) patients, mild cognitive impairment (MCI) patients and Alzheimer’s disease (AD) patients. We propose a new framework to study the topological networks with a spatiotemporal entropy measure for estimating the connectivity. Our results show that functional connectivity and graph analysis are frequency-band dependent, and alterations start at the MCI stage. In delta, the SCI group exhibited a decrease of clustering coefficient and an increase of path length compared to MCI and AD. In alpha, the opposite behavior appeared, suggesting a rapid and high efficiency in information transmission across the SCI network. Modularity analysis showed that electrodes of the same brain region were distributed over several modules, and some obtained modules in SCI were extended from anterior to posterior regions. These results demonstrate that the SCI network was more resilient to neuronal damage compared to that of MCI and even more compared to that of AD. Finally, we confirm that MCI is a transitional stage between SCI and AD, with a predominance of high-strength intrinsic connectivity, which may reflect the compensatory response to the neuronal damage occurring early in the disease process.

## 1. Introduction

The human brain is a highly complex self-organizing system. Its functioning relies on the collective dynamics of millions of neurons interconnected through a sophisticated network of synapses that are well organized in their structure and connectivity. Synaptic dysfunction has received significant attention, particularly since there has been evidence that the loss of neuronal synapses occurs in the early stage of neurodegenerative diseases (NDD) [1]. Recent research suggested that synapses are sites of aberrant protein misfolding in NDD [2].

Alzheimer’s disease (AD) is the most prevalent NDD, which accounts for 50% to 70% of cases [3]. It is a chronic and insidious disease that produces a progressive cognitive decline. There is a growing interest in earlier stages due to the lack of curative treatments. The preclinical stage is asymptomatic but the brain lesions of AD are present. At this stage, the term of Subjective Cognitive Impairment (SCI) is defined by a self-experienced persistent decline in cognitive capacity compared to a normal status [4,5,6]. 

In the Mild Cognitive Impairment (MCI) stage, patients exhibit measurable memory impairments but maintain their functional capacities [5,7,8]. SCI and MCI patients are at risk of developing AD [5,6]; an in-depth understanding of the mechanisms involved in these early stages of AD is thus crucial. Functional Magnetic Resonance Image (fMRI) is a largely used brain imaging technique, which provides a sequence of images of brain activity by measuring the cerebral blood flow. However, it is employed for AD diagnosis at the price of a high cost. The captured images are also static, making the fMRI unsuitable to follow brain dynamics. 

Electroencephalography (EEG) has been considered as a convenient technique in clinical applications that is suitable for cognitively and physically disabled patients, as well as for serial tests in absence of objective cognitive impairment [9]. EEG has the advantage of being a non-invasive, cost-effective and widely available brain imaging technology. 

In addition, EEG signals are characterized by a high temporal resolution, which is crucial for the analysis of fast brain functional dynamics at different frequency ranges (1–4 Hz, delta; 4–8 Hz theta; 8–12 Hz, alpha; 12–30 Hz, beta; and >30 Hz, gamma). On the other hand, various studies in the literature demonstrated the potential use of EEG signals to identify different abnormal brain conditions, including depression [10,11,12], seizures [13,14,15] and NDD, such as Parkinson’s disease [16,17,18] and AD [9,18,19,20,21].

Several studies highlighted changes in EEG signals recorded in eyes-closed resting-state condition (rsEEG) at the early stage of AD. For the purpose of AD diagnosis, three branches of EEG signal analysis have emerged: spectral, complexity and functional connectivity analyses. Pioneering spectral analysis reported an increased activity in delta and theta bands, as well as decreased activity in alpha and beta bands in AD patients [9,19,20,21,22,23,24,25], thus, suggesting a slowing of EEG signals. Numerous studies have revealed that AD induces a reduction in complexity of the EEG signal compared to that of healthy subjects [25,26,27,28,29,30,31,32]. 

Other studies investigated functional connectivity between electrode pairs [24,25,33,34] to assess the degree of signal synchronization between different brain areas, using a large variety of measures, such as the phase-lag index [35,36], coherence [37,38,39,40], mutual information [41] and synchronization likelihood [42,43]. Note that two locations are functionally connected if they have coherent or synchronized dynamics in their captured EEG signals. The majority of these studies reported a loss of functional connectivity in AD and MCI compared to healthy controls (HC) in high-frequency ranges, especially in the alpha band. Delta and theta functional connectivity measurements provided less straightforward outcomes [28,29,37]. 

More recently, graph theory [44,45,46,47,48,49] has gained considerable ground in investigating topological differences between normal and abnormal brain networks, especially in the context of AD dementia. By modeling a brain network as a graph involving nodes (electrodes) interconnected by edges that represent the connectivity between cortical nodes, it is possible to conduct a topological analysis of the brain functional organization. Of note, functional connections in the network correspond to statistical relationship between EEG signals rather than physical linkages and, therefore, do not relate to direct metabolic events. 

Various topological parameters are applied to characterize the network. Most prominently, the “clustering coefficient” characterizes the tendency of a network to form small clusters of closely interconnected nodes, and the “shortest path” characterizes the global efficiency of information transfer within the network. The “small-world” network structure reflects an optimal balance of efficient information transmission between long-range connections (small path length), while maintaining efficient local information processing (high clustering coefficient). By contrast, “random” structures tend to have a long average path length and a low clustering coefficient.

Several studies have reported that the network topology is altered in AD and MCI patients compared to HC [47,48,50,51,52,53,54,55,56,57,58,59,60,61,62,63,64,65,66,67,68,69,70,71]. Studies have shown that the AD group deviates from the optimal small-world topology towards a more random one compared to HC [50,51,52]. For the other topological parameters, contradictory results have been observed in the literature [50,51,52,53,54,55,57,65,67]. This is mainly due to the use of sparse data sets with different characteristics as well as methodological differences. 

More precisely, EEG databases with different characteristics are used in the literature for comparing AD patients to HC and MCI. In addition, they are prone to experimental constraints that do not match the reality on the ground, such as including strict patient inclusion and exclusion criteria and considering normal healthy subjects as controls. 

In addition, most studies consider undirected binary networks, which require the application of an arbitrary threshold on adjacency connectivity matrices. This factor affects directly the resulting network. The choice of using weighted or binary matrices to estimate network graph has mostly been arbitrary to date. Recently, some studies addressing this issue [65,66] found that preserving real-valued weights produces consistent results by exploiting the additional topological information that is stored in the weights.

Moreover, several measures can be exploited to quantify the connectivity in brain networks. These measures may reflect different processes, which lead to different network topologies. The majority of studies used a specific measure without comparing it to others on the same database and without studying the contribution of graph analysis with respect to classical connectivity analysis. The variability in calculation routines leads to difficulty in comparing the results between studies that are sometimes contradictory.

In addition, all graph-based studies characterized links in graph networks using only the degree of signal synchronization between electrodes without considering the other EEG abnormalities in AD, such as a reduction of EEG complexity. More precisely, the traditional functional connectivity measures share two main drawbacks. First, they quantify the spatial relationship between EEG time series and do not consider the complete spatiotemporal alterations due to AD, namely the reduction of both complexity and inter-channel connectivity. Second, they neglect the physiological reality that the brain’s information processing is not stationary and that spontaneous transitions occur even in the resting-state condition.

By contrast to the above-mentioned investigations, the present study relates to the analysis of the functional connectivity network in SCI, MCI and AD stages, based on rsEEG data acquired in real-life clinical conditions. To this aim, we exploited a specific spatiotemporal connectivity measure, called Epoch-based Entropy (*EpEn*) [72,73,74,75], to perform a weighted graph analysis of SCI, MCI and AD brain networks. 

*EpEn* stems from a refined characterization of the local statistical properties of EEG signals using continuous Hidden Markov Models (HMM). It has been shown in previous works [73,74,75] that this modeling approach, combined to *EpEn*, is suitable to the analysis of the underlying neuronal dynamics in the context of AD, since it quantifies, on piecewise stationary epochs, the information content conveyed by EEG signals locally over time (as done by classical complexity measures) and also spatially by estimating inter-channel relationships. 

In previous works, such measures were computed per brain region on a set of EEG signals belonging to the considered region [72,73,74,75]; in this paper, we propose to compute *EpEn* on all possible pairwise electrodes for a refined characterization of the functional connectivity, which will be exploited for network topology analysis. 

By means of graph theory analysis on the obtained adjacency matrices, we assess the hypothesis that the refined characterization of EEG signals based on our statistical spatiotemporal entropy measure combined to the topological characterization of the brain network, could allow a better understanding of the global connectivity organization between SCI, MCI and AD populations.

The novelty of the paper is twofold. First, this study aims at analyzing EEG brain network over SCI, MCI and AD stages simultaneously and contributes to providing a deep interpretation of our findings considering different graph parameters and frequency bands. Indeed, our objective is to make the obtained results more understandable and pertinent, particularly for clinicians. 

Second, to our knowledge, this is the first study combining an entropy-based measure to graph theory considering a weighted network analysis. The advantage of the entropy measure consists in the integration of the complete spatiotemporal alterations due to AD. We show that this new framework allows conducting a refined brain network analysis, which highly contributes to a better understanding of the evolution of AD from SCI to dementia through the MCI stage. In a more recent study [76], we combined *EpEn* to graph theory but considered binary network analysis to discriminate automatically between SCI, MCI and AD patients (a classification problem). By contrast, the present work investigates the brain network topology over the three stages in order to retrieve global patterns that characterize the evolution towards dementia.

## 2. Materials and Methods

The present study was conducted on a cohort containing rsEEG data of three populations (SCI, MCI and AD patients) recorded in real-life clinical conditions. In order to investigate the differences between SCI, MCI and AD, we first studied the functional connectivity in the three groups using the entropy measure (*EpEn*). To assess the effectiveness of our entropy metric, we compared it to two alternative measures, commonly used in the literature: the Magnitude Square Coherence (MSC) and Phase Lag Index (PLI). Then, by means of graph theory analysis on the obtained *EpEn* matrices, we studied the organizational properties of brain networks in SCI, MCI and AD. 

Later, we describe the EEG database used and present the three connectivity measures as well as the topological parameters used for the graph analysis. 

### 2.1. Study Population

The cohort contains rsEEG data of 102 subjects recorded in real-life clinical conditions at the Charles-Foix Hospital in France. Subjects who complained of memory impairment were referred to the outpatient memory clinic of the hospital to undergo a battery of clinical and neuropsychological tests for brain disorders. 

For each subject, a diagnosis was established at the memory clinic on the basis of the clinical assessment, brain imaging, psychometric findings, interviews and neuropsychological tests, conducted by a multi-disciplinary medical staff, according to the standard diagnostic criteria: DSM-IV, NINDS, Jessen criteria for SCI and Mc Keith criteria for Lewy body dementia [4,5,77]. 

Patients with epilepsy were excluded, and EEG was not used to establish the diagnosis. This retrospective study was approved by the institutional review board of the local Ethics Committee Paris 6, on 16 May 2013. Before conducting this retrospective study, an information letter on the research study was sent to the patients, detailing the possibility of opposing the use of their collected data. All the data were fully anonymized before use in our research.

The study population includes rsEEG recordings of 22 SCI subjects, 52 MCI patients and 28 mild to moderate AD patients. Table 1 reports information about the demographic and clinical characteristics of the patients.

### 2.2. EEG Recording and Preprocessing

EEG data were recorded during the resting state eyes-closed condition using a Deltamed digital EEG acquisition system with 30 scalp electrodes positioned over the whole head according to the 10–20 international system: Fp1, Fp2, F7, F3, Fz, F4, F8, FT7, FC3, FCz, FC4, FT8, T3, C3, Cz, C4, T4, TP7, CP3, CPz, CP4, TP8, T5, P3, Pz, P4, T6, O1, Oz and O2. All data were digitalized in a continuous recording mode for a minimum of 20 min with a 256 Hz sampling frequency.

The EEG recordings were pre-processed off-line on MATLAB software (MathWorks Inc., Natick, MA, USA). For each subject in the database, continuous epochs of 20 s, free from artifacts (eye movements, eye blinks, muscular activity, instrumental noise etc.), were manually selected. To do that, an EEG expert visually inspected the EEG signals and discarded the parts of the signals presenting artifacts. The extracted clean 20-s segments were then kept for the study. Note that the EEG expert was blinded from the results of the present study.

Then, the obtained free artifact 20-s EEG signals were notch filtered at 50 Hz to eliminate possible artifacts caused by power line interference. Finally, the obtained EEG signals were band pass filtered with a third-order digital Butterworth filter in the four conventional frequency bands of interest, i.e., delta (1–4 Hz), theta (4–8 Hz), alpha (8–12 Hz) and beta (12–30 Hz).

### 2.3. Functional Connectivity Measures

#### 2.3.1. Phase Lag Index

The Phase Lag Index (PLI) measures consistency across time of the instantaneous delay between two time series. It is largely used with EEG due to its robustness to head volume conduction, which is a common issue in EEG recordings [9,34,35,36].

PLI is based on the asymmetry of the distribution of instantaneous signal phase differences. It relies on the idea that a consistent non-zero phase difference (phase lag) reflects a time lag between two EEG signals [35,36]. The main approach is to neglect phase differences that are centered around 0 mod π [36]. The index of the asymmetry of the phase difference distribution is calculated as:

(1) $$\mathrm{PLI}=\left|mean\left(sign[\sin\left(\Delta \emptyset \left(t_{k}\right)\right]\right)\right|$$

where Δ∅ is the phase difference at time $t_{k}\;$between two time series, calculated for all time-points per epoch; sign stands for signum function.

The PLI ranges between 0 and 1. A zero value indicates either no coupling or coupling with a phase difference centered around 0 mod π. A PLI equals to 1 indicates a perfect phase locking at a value of Δ∅. The higher is this nonzero phase locking, the higher is the PLI.

#### 2.3.2. Magnitude Square Coherence

The coherence measure captures the linear component of the functional coupling of the paired EEG oscillations *x* and *y* as a function of a frequency f [33,37,38,39,40]. In order to compute the magnitude square coherence (MSC), signals *x* and *y* are subdivided in M segments of equal length L, and then the coherence function is computed by averaging over those segments. The magnitude square coherence c(f) is calculated as:

(2) $$c\left(f\right)=\frac{{\left|\langle X\left(f\right)Y^{*}\left(f\right)\rangle \right|}^{2}}{\left|\langle X\left(f\right)\rangle \right|\left|\langle Y\left(f\right)\rangle \right|}$$

where X(f) and Y(f) are the Fourier transforms of *x* and *y*, respectively; $Y^{*}$ is the complex conjugate of Y; |.| denotes the magnitude value, and 〈.〉 denotes the mean value computed over the M segments.

The MSC ranges between 0 and 1. A high coherence between two EEG signals indicates an efficient communication (a high connectivity) between the two electrodes that captured such signals.

#### 2.3.3. Epoch-Based Entropy

The epoch-based entropy measure (*EpEn*) was introduced in [72,73] for early AD screening. It estimates the complexity of EEG signals not only locally over time (as classical complexity measures do) but also spatially by estimating the inter-channel complexity. This statistical measure quantifies the information content or the disorder inherited in the considered signal or conveyed by a couple of EEG signals.

**Figure 1:**
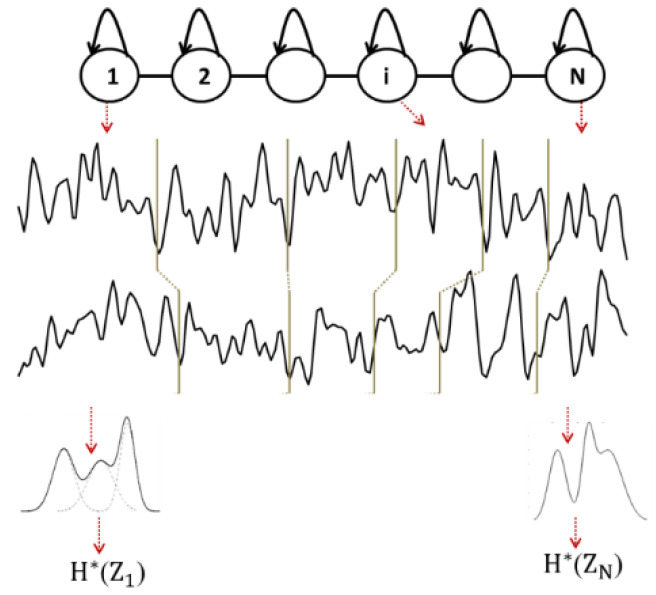
Illustration of multi-channel (*N* = 6, *D* = 2) EEG signal modeling with HMM [69].

*EpEn* is computed on piecewise stationary epochs of EEG signal using a Hidden Markov Model (HMM), which performs a local density estimation at the epoch level. As in our previous studies [72,73,74,75], EEG signals are modeled by a continuous left-to-right HMM (Figure 1). The states of the HMM correspond to the stationary epochs of the EEG signal, and the transitions of the HMM correspond to the variations of the signal. 

The EEG signal recorded from a given subject is thus considered as a succession of epochs, obtained by segmenting the signal by the Viterbi algorithm using the corresponding subject’s HMM. Thus, each obtained epoch corresponds to a state of the HMM and contains a given number of observations (sample points). For each epoch $S_{i},$ the probability density function is modeled by a mixture of M Gaussian functions (Figure 1).

Then, each observation z contained in one epoch $S_{i}\;$is considered as a realization $Z_{i}$ of a random variable Z, which follows a given observation probability distribution$\;P_{i}\left(z\right)$ modeled by the Gaussian mixture. Each stationary epoch of the signal is then associated to a random variable, and the entropy $H^{*}\left(Z_{i}\right)\;$of the epoch $S_{i\;}$is that of an ensemble of realizations of$\;Z_{i}$:

(3) $$\;H^{*}\left(Z_{i}\right)=-\underset{z\in S_{i}}{\sum }P_{i}\left(z\right)\times {log}_{2}P_{i}\left(z\right)$$

By averaging the entropy over the *N* epochs of the EEG signal of the subject, an entropy value EpEn(Z) of the signal is obtained:

(4) $$EpEn\left(Z\right)=\frac{1}{N}\overset{N}{\underset{i=1}{\sum }}H^{*}(Z_{i})$$

To model the inter-relations between two EEG time series recorded from two cortical electrodes, an HMM is trained for each subject on such couple of EEG signals. At time t, a hidden state emits a two-dimensional observation vector. By applying the Viterbi algorithm, each EEG signal is segmented into *N* epochs, and the entropy $H^{*}\left(Z_{i}\right)\;$of each epoch $S_{i\;}$ is computed considering the probability density estimated by the HMM on the observations of the *N* epochs (Figure 1). 

Although all *N* epochs are matched between EEG channels, the model does not constrain these epochs to be of equal length. Finally, by averaging the entropy over all the *N* epochs, an epoch-based entropy value (*EpEn*) associated to the multi-channel EEG of the subject is computed. A high value of *EpEn* indicates a high information content conveyed by the coupling of two EEG signals.

### 2.4. Brain Network Analysis

In the present study, we conducted brain network analysis on weighted and fully connected functional connectivity matrices. In other words, the adjacency connectivity matrices, which are real-valued, were not thresholded to preserve all the available information.

In the following, we provide mathematical definitions in the weighted framework of the three commonly used and complementary topological parameters: the clustering coefficient, shortest path and modularity. In addition, we propose a precise interpretation of such parameters suitable to the new framework brought by our study. 

#### 2.4.1. Clustering Coefficient

The clustering coefficient of a node evaluates the density of connections formed by its neighbors [46,47,49]. If a node i has $k_{i}$ neighbors, the weighted clustering coefficient *C* of node i is defined as:

(5) $$C_{i}=\frac{2\sum_{j,k}{\left(w_{ij}w_{ik}w_{jk}\right)}^{1/3}\;}{k_{i}\left(k_{i}-1\right)}$$

where $w_{ij}$ is the connectivity weight between nodes i and j, and $k_{i}$ is the number of connections in node i.

A high value of the local clustering coefficient$\;C_{i}$ indicates that the neighbors of a node i that present high strength connectivity are densely interconnected. The clustering coefficient is often associated to a measure of segregation: this reflects the tendency of a network to form topologically local densely circuits (cliques) presenting high-strength intrinsic connectivity.

#### 2.4.2. Shortest Path

The shortest weighted path is a parameter of integration, which quantifies how the information is exchanged or integrated in the whole brain network [46,47,49]. A path is any sequence of edges that connects two nodes, and its length is given by the sum of the connection weights. The shortest weighted path length $d_{i,j}^{w}\;$between node 𝑖 and 𝑗 is defined as:

(6) $$d_{i,j}^{w}=\underset{w_{ij}\in gi⟷j}{\sum }w_{ij}$$

where gi⟷j is the shortest weighted path between nodes 𝑖 and 𝑗. 

The weighted path length *L* at node *i* is defined as:

(7) $$\;L_{i}=\frac{\sum_{i\neq j}d_{i,j}^{w}}{\left(n-1\right)}$$

where *n* is the number of nodes (*n* = 30 in our study) and $d_{i,j}^{w}$ is the shortest path length between nodes i and j, considering all possible paths that have to be spanned from node i to node j.

A low value of edges in the shortest path suggests that information is routed between electrodes with few intermediate steps (edges), which indicates rapid and high efficiency in the information transmission across the network.

#### 2.4.3. Modularity

The modularity index reveals a hierarchical structure of a graph network, decomposed into densely intra-connected groups of nodes (modules) that are sparsely inter-connected with nodes in other modules of the network [46,47,49].

This modular structure is organized hierarchically, such that it contains sub-modules over several topological resolution scales. This organization can be consistent with a fractal community structure. The modular structure, subdividing the network into non-overlapping subnetworks (modules), is achieved by searching for the partition with a maximally possible number of within links and a minimally possible number of links between modules. The optimal modular structure is typically estimated with an optimization algorithm [78], which aims at maximizing the following quantity:

(8) $$Q=\frac{1}{L}\underset{ij}{\sum }\left[w_{ij}-\frac{k_{i}k_{j}}{L}\right]\delta_{m_{i}m_{j}}\;$$

where $w_{ij}\;$ is the connection strength (weight) between nodes i and j; $k_{i}\;$ is the number of connections in node i; L is the weighted characteristic path length; and $\delta_{m_{i}m_{j}}\;$ is equal to one if nodes i and j belong to the same module and zero otherwise. This ensures that we only count edges between nodes within the same module. 

Modularity is a general hallmark of complex biological systems. It highlights flexibility and adaptability. Modular architecture naturally arises in networks that can adapt and evolve to changing environmental events, such as the onset of pathology. 

### 2.5. Statistical Analysis

Statistical analyses were performed using MATLAB R2020a software. We compared characteristics between the SCI, MCI and AD groups using the Kruskal–Wallis test. This statistical test is a nonparametric version of the one-way ANOVA and is an extension of the Wilcoxon rank sum test to more than two groups. The results with a p-value lower than 0.05 are considered to be statistically significant. To evaluate the differences among the three groups in terms of functional connectivity measures, the statistical test was applied in Section 3.1 on each of the 30 electrodes and at each frequency band. 

We also assessed the significant difference between groups in terms of the graph parameters computed on the average connectivity matrices. The Kruskal–Wallis test was performed on each frequency band considering, for each group, the 30 clustering coefficient values associated to the 30 electrodes (Section 3.2). For the shortest path comparisons, the statistical analysis between the three groups was conducted on each electrode and each frequency band (Section 3.3). 

## 3. Results

### 3.1. Functional Connectivity Assessment with Three Metrics

**Figure 2:**
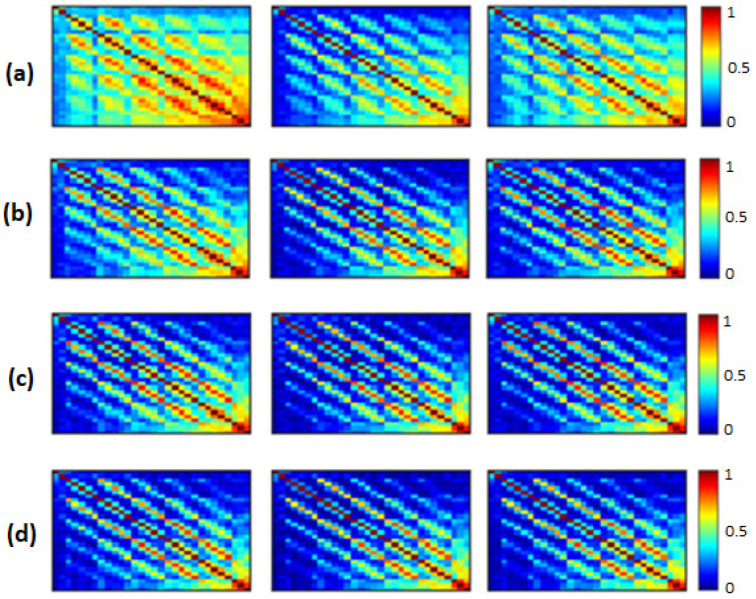
The average MSC across subjects in the SCI (left), MCI (middle) and AD (right) groups for the (**a**) delta, (**b**) theta, (**c**) alpha and (**d**) beta bands. The 30 electrodes are shown from left to right side, anterior-posteriorly (up to bottom). The color bar indicates the values of MSC.

**Figure 3:**
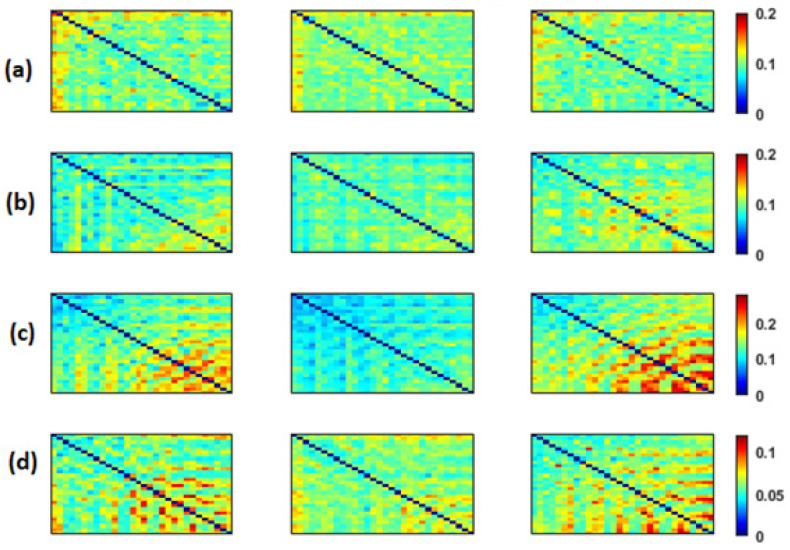
The average PLI across subjects in the SCI (left), MCI (middle) and AD (right) groups for the (**a**) delta, (**b**) theta, (**c**) alpha and (**d**) beta bands. The 30 electrodes are shown from left to right side, anterior-posteriorly (up to bottom). The color bar indicates the values of PLI.

**Figure 4:**
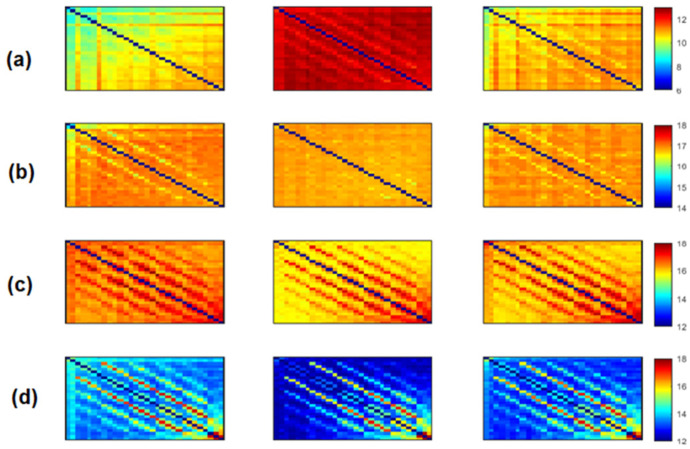
Average *EpEn* across subjects in the SCI (left), MCI (middle) and AD (right) groups for the (**a**) delta, (**b**) theta, (**c**) alpha and (**d**) beta bands. The 30 electrodes are shown from left to right side, anterior-posteriorly (up to bottom). The color bar indicates the values of *EpEn*.

We computed the three functional connectivity measures between all pairs of the 30 electrodes for each person, and then averaged across subjects of the SCI, MCI and AD groups. Figure 2, Figure 3 and Figure 4 show the average adjacency matrices (30*30) with MSC, PLI and *EpEn*, respectively, for the three populations in the four frequency bands. The electrodes are positioned on the matrices from left to right, anterior-posteriorly, as follows: Fp1, Fp2, F7, F3, Fz, F4, F8, FT7, FC3, FCz, FC4, FT8, T3, C3, Cz, C4, T4, TP7, CP3, CPz, CP4, TP8, T5, P3, Pz, P4, T6, O1, Oz and O2. The diagonal elements of such matrices represent the connectivity of each electrode with itself. Thus, we ignored the diagonal elements in our analysis to consider only pairwise dynamics.

We visually notice that Figure 2, Figure 3 and Figure 4 exhibit different patterns in the average adjacency matrices between SCI, MCI and AD as a function of the functional connectivity measure and the frequency band. For a precise comparison between the three groups, we performed the Kruskal–Wallis test to detect significant differences between matrices on each electrode and at each frequency band as presented in Section 2.5.

We noticed a significant difference between the SCI, MCI and AD groups with MSC in the delta band for all electrodes (p<0.05) except TP7, in the theta band at {FP1, FP2, F7, FT7, FC3, FCz, T3, CP3, CPz, P4, T6} and in the beta band at {FT7, CP3}. No significant difference was observed with MSC in the alpha band. Concerning PLI, we observed a significant difference between the three groups in delta at {Fp1, Fp2, F3, FT7, CP3, CPz, TP8, T5, P4, O1, Oz}, in theta at several electrodes except for {Fp2, F8, FT8, C3, C4, T4, TPz, TP8, T5, P3, P4, O2}, in the alpha band at all electrodes and in the beta band at { Fp1, Fp2, FT7, CP3, T6}.

Regarding *EpEn* measure, Figure 4 shows a clear difference in the global functional connectivity organization between the three groups in all frequency bands. In delta, a significant difference is obtained between the three groups at all electrodes ($p<1\times 10^{-12}$). In addition, in Figure 4a, we clearly observe a very high information content in MCI between all pairs of electrodes, which reduces in AD and even more in SCI, especially in the prefrontal and frontal regions. In theta, the difference between the three groups is less pronounced visually (Figure 4b); however, the statistical analysis revealed a significant difference at all electrodes, except at {F3, P4}.

In high frequencies (Figure 4c,d), the information content between all pairs of electrodes quantified by *EpEn* is higher for SCI and reduces for AD and even more for MCI. A significant difference between the three groups (p<0.05) is observed in the alpha and beta bands at all electrodes.

**Figure 5:**
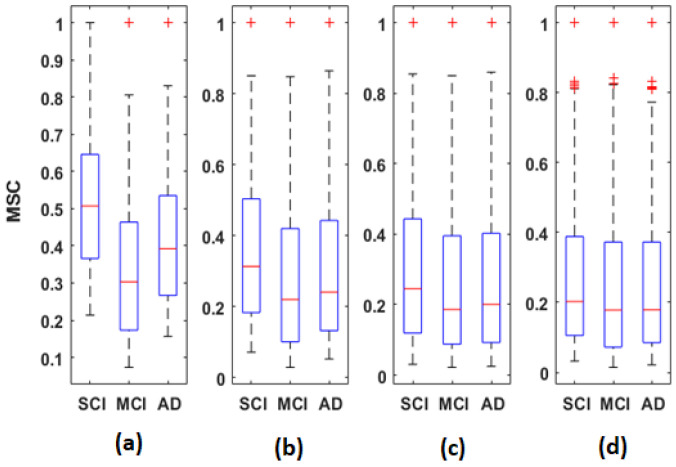
Boxplots of MSC values averaged over all subjects of the SCI, MCI and AD groups on the (**a**) delta, (**b**) theta, (**c**) alpha and (**d**) beta bands.

**Figure 6:**
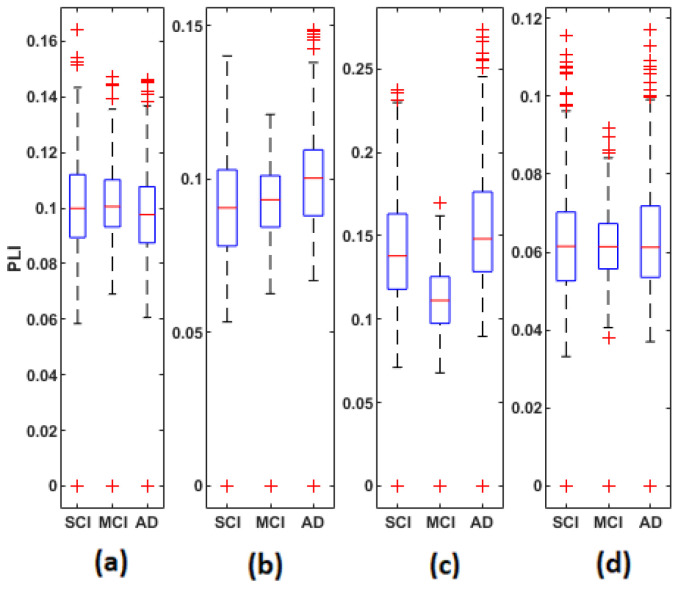
Boxplots of PLI values averaged over all subjects of the SCI, MCI and AD groups on the (**a**) delta, (**b**) theta, (**c**) alpha and (**d**) beta bands.

**Figure 7:**
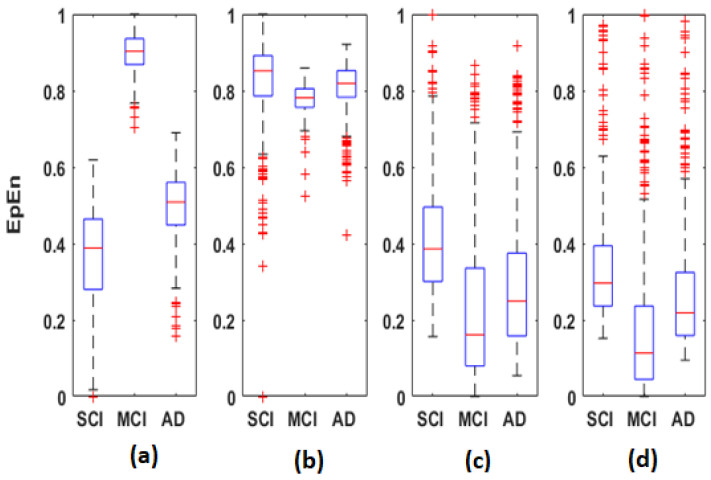
Boxplots of *EpEn* values averaged over all subjects of the SCI, MCI and AD groups on the (**a**) delta, (**b**) theta, (**c**) alpha and (**d**) beta bands.

Figure 5, Figure 6 and Figure 7 display the distribution of the connectivity values computed between all pairs of the 30 electrodes, for the three groups in the four frequency bands. These connectivity values (435 values) correspond to those of the upper triangular part of the average matrices in Figure 2, Figure 3 and Figure 4, since these matrices are symmetric. 

Overall, the statistical spatiotemporal entropy measure appears as the EEG marker, which highlights a better distinction between the three groups compared to the two other deterministic measures. The contrast between SCI, MCI and AD is observed with *EpEn* at different frequency bands and almost all electrodes. When comparing *EpEn* values between AD and SCI, the AD group presents higher values in delta and lower values in alpha and beta (Figure 7). This result is in accordance with previously published studies: AD leads to an increased activity in delta and decreased activity in alpha and beta bands [9,19,20,21,22,23,24,25].

Regarding the MCI group, it exhibits the highest *EpEn* values in delta and the lowest values in alpha and beta. The MCI group shows a more accentuated behavior compared with AD relatively to SCI. In theta, the difference between the three groups with *EpEn* is less pronounced compared to the other frequency bands.

**Figure 8:**
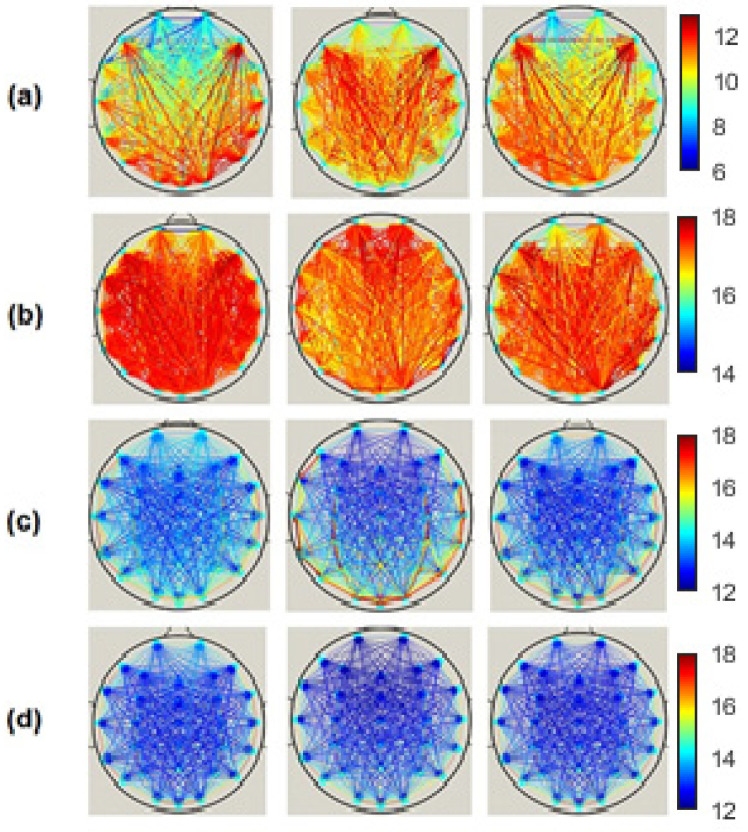
Connectivity network of the SCI (left), MCI (middle) and AD (right) groups on the (**a**) delta, (**b**) theta, (**c**) alpha and (**d**) beta bands.

*EpEn* shows more differentiation between the three cognitive decline stages, at all electrodes, compared to the two other classical measures. Since we exploit the local clustering coefficient and local shortest path parameters, in the rest of the paper, we investigate brain networks of SCI, MCI and AD using only *EpEn* to represent the connectivity between nodes. Figure 8 illustrates the network connectivity between the 30 electrodes using the *EpEn* measure.

### 3.2. Clustering Coefficient

**Figure 9:**
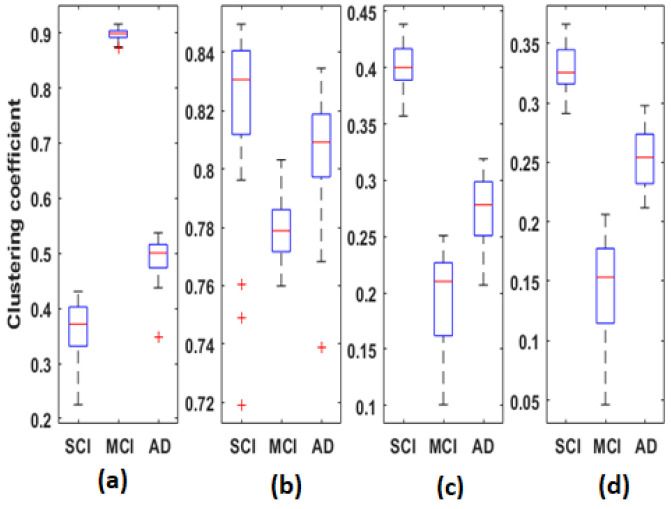
Boxplots of the local clustering coefficient values computed for each average *EpEn* matrix of SCI, MCI and AD on the (**a**) delta, (**b**) theta, (**c**) alpha and (**d**) beta bands.

Figure 9 represents the distribution of clustering coefficient values computed for each average *EpEn* matrix of each group, in the four frequency bands. Each boxplot contains 30 local clustering coefficient values associated to the 30 nodes (electrodes).

When comparing Figure 7 and Figure 9, we notice that the clustering coefficient is correlated to *EpEn*: the relative positioning of the three populations, in the four frequency bands, is almost similar. Nevertheless, the clustering coefficient leads to a better characterization of the three populations compared with *EpEn*. The difference between SCI, MCI and AD is significant ($p<1\times 10^{-10})$ for all frequency bands.

In theta, alpha and beta, the clustering coefficient presents the same tendency across the three groups; the opposite behavior is observed in delta.

**Figure 10:**
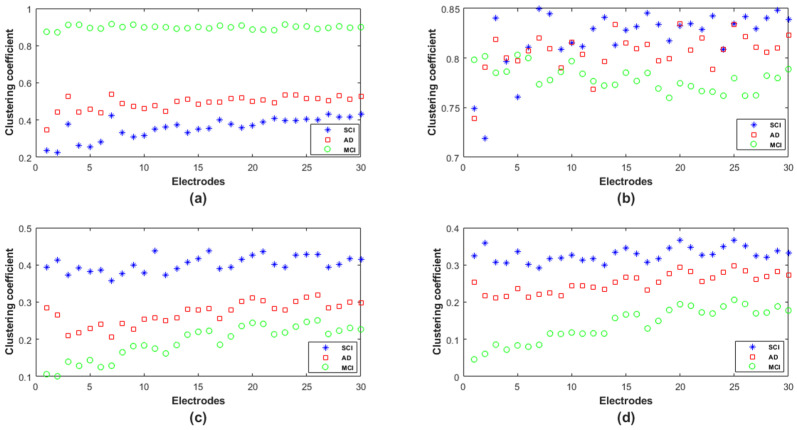
Clustering coefficient values over the 30 electrodes for SCI, MCI and AD on the (**a**) delta, (**b**) theta, (**c**) alpha and (**d**) beta bands.

In order to provide deeper insights regarding the topological organization in SCI, MCI and AD in terms of the clustering coefficient, we analyzed such parameters locally at each electrode (Figure 10). We notice that, except for theta, there was no overlap between the three populations in delta, alpha and beta; however, the behavior of this graph parameter across the three populations was frequency-band-dependent. 

In delta, the MCI group shows the highest values of the clustering coefficient for all nodes; the SCI group shows the lowest values. The opposite is observed in the other frequency ranges, especially alpha and beta. Finally, the clustering coefficient values of AD group are in between those of SCI and MCI, regardless of the frequency band.

Therefore, it is clear that the local clustering coefficient allowed a better separation between SCI, MCI and AD and, thus, a better characterization of the three stages of the disease.

### 3.3. Shortest Path

**Figure 11:**
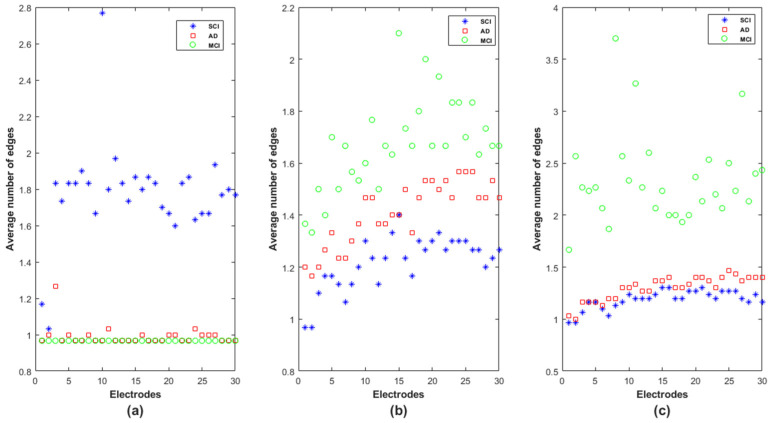
Average number of edges in the shortest path at each node for SCI, MCI and AD on the (**a**) delta, (**b**) alpha, and (**c**) beta bands.

At each node, we computed all the shortest paths between the node and the other 29 nodes (as in Equation (6)). For each shortest path, we calculated the number of edges composing the obtained path. Figure 11 reports the average number of edges in the obtained shortest paths at each node for the SCI, MCI and AD groups in delta, alpha and beta. In theta, the results do not show a difference between the three groups in terms of the number of edges in each electrode as confirmed by the statistical test conducted on each frequency band and each electrode (p>0.05). 

Note that the electrodes are positioned in Figure 11 following the same order as in the adjacency matrices: Fp1, Fp2, F7, F3, Fz, F4, F8, FT7, FC3, FCz, FC4, FT8, T3, C3, Cz, C4, T4, TP7, CP3, CPz, CP4, TP8, T5, P3, Pz, P4, T6, O1, Oz and O2. We recall that a low value of edges in the shortest path indicates that information exchange is rapid and efficient in the whole network. 

In delta, the SCI group is, by far, the one with the longest shortest path in terms of intermediate steps (Figure 11a). The MCI and AD groups are almost similar, except on F7 (electrode *n*°3), for which the MCI group shows the lowest average shortest path, which increases on AD and even more on SCI. The opposite behavior appears in the alpha and beta bands: the MCI and SCI groups exhibit, respectively, the highest and the lowest values of average shortest path; the AD group is in between. However, we notice that the separation between the three groups is better in the alpha band on almost all nodes (Figure 11b).

For a better understanding of the functioning of this graph parameter, we observed the shortest path for the SCI group was in delta between F7 and FCz. We found that the shortest path was not the direct link between such electrodes, as was the case for AD and MCI. The information was instead routed via two other nodes Fp2 and Fz (F7 → Fp2 → Fz → FCz). This result reveals that many short-term connections are set up in SCI to transmit the information between two electrodes in the delta band. 

In addition, regarding the shortest path of the MCI group on beta, in order to transmit the information from FT7 to FT8, the brain network displays the following path FT7 → FC4 → T3 → Fz → T4 → F3 → FT8; while the direct connection between FT7 and FT8 is observed for the SCI group. This result suggests that the MCI group exhibits many short-term connections for information exchange at high frequencies.

### 3.4. Modularity

**Figure 12:**
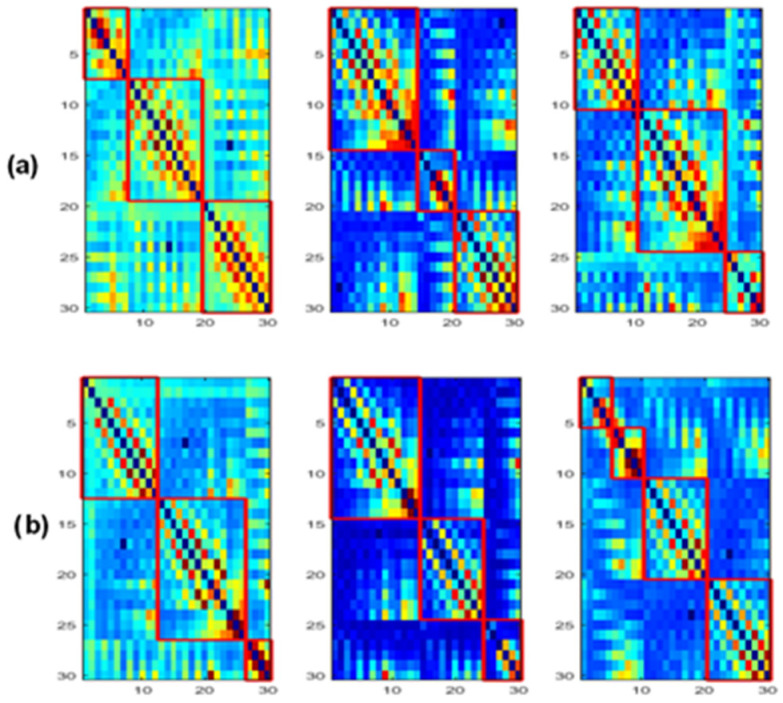
The obtained modules for SCI (left), MCI (middle) and AD (right) on the (**a**) alpha and (**b**) beta bands.

Figure 12 shows the obtained modules in alpha and beta for the three populations. In delta and theta, the results showed that all electrodes belong to only one module (*Q* = 1), meaning that there is an absence of sub-networks (no modular structure) in our framework that considers fully connected matrices, i.e., all the connections in the network are maintained. Note that a sub-network refers to a group of nodes having denser relations with each other than with the rest of the network.

**Figure 13:**
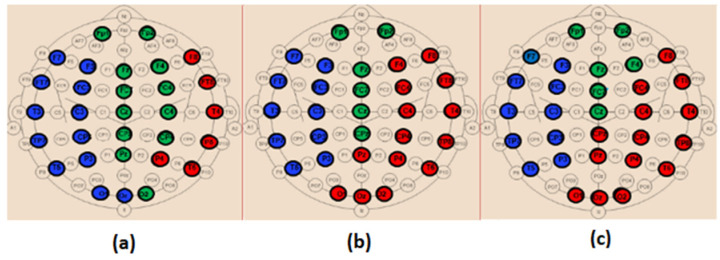
The distribution of the nodes in each module in the alpha band for the (**a**) SCI, (**b**) MCI and (**c**) AD groups.

**Figure 14:**
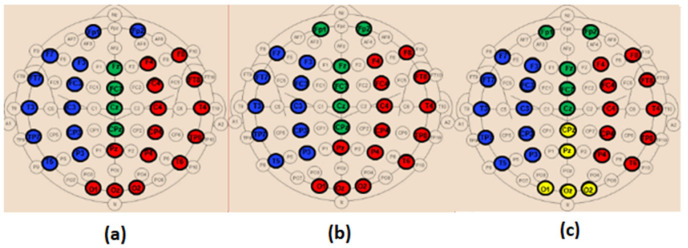
The distribution of the nodes in each module in the beta band for the (**a**) SCI, (**b**) MCI and (**c**) AD groups.

Figure 13 and Figure 14 display the composition of the obtained modules in the three populations, in alpha and beta, respectively.

When comparing the spatial distribution of the obtained modules in the alpha band for SCI and AD groups (Figure 13a,c), we first notice that two modules among the three ones observed in SCI extend from anterior to posterior regions. More precisely, in Figure 13a, the module in green contains electrodes from the prefrontal {FP1, FP2} to occipital {O2} regions passing by the frontal, central and parietal regions. The blue one contains electrodes from the frontal to occipital regions. In addition, we notice that the electrodes of the same brain region are distributed over several modules, particularly electrodes of the occipital region. 

These findings may indicate a strong dynamic interaction between different brain areas for SCI group, which facilitates the long-term information transmission. Indeed, the interaction between electrodes belonging to the same module is relatively strong according to the definition of the modularity. More precisely, we observe a clear difference between SCI and AD in the occipital area (Figure 13). 

The SCI group presents a strong interaction between the occipital region and the left anterior regions since {O1, Oz} exist in the same module as {F7, F3, FT7, FC3, T3, C3, TP7, CP3, T5, P3}. In parallel, {O2} exists in the same module as {Fp1, Fp2, Fz, FCz, CPz, Pz, F4, FC4, C4, CP4}, and thus the occipital region has also a strong interaction with anterior regions by another way. This is not the case for the AD group, where the interaction of the occipital region is limited to the right area of the brain (Figure 13c). In addition, the prefrontal region in AD interacts only with some electrodes from the frontal and central regions {Fz, F4, FCz, Cz}. 

In beta, we also observe a stronger long-term connectivity between brain regions for SCI compared to AD (Figure 14). For the SCI group (Figure 14a), the occipital region has a strong connectivity with the right brain area, including the parietal, central, temporal and frontal electrodes. However, for the AD group (Figure 14c), the occipital electrodes {O1, Oz, O2} are grouped in one module with Pz and CPz, which reflects the weak interaction of the occipital area with the other regions.

Regarding the MCI group, their topology in the alpha band is closer to that of AD compared to SCI (Figure 13b). In the beta band, the topology of MCI (Figure 14b) is closer to that of SCI; nevertheless, the prefrontal region {Fp1, Fp2} has a relatively weak connectivity with the left part of the brain as for AD. These results support the fact that MCI group has an intermediate behavior between the SCI and AD groups.

## 4. Discussion

Previous rsEEG studies on functional organization of the brain network in the context of AD reported conflicting results [50,51,52,53,54,55,57,65,67]. These discrepancies among studies could be related to methodological differences and the use of databases with different characteristics, which are sometimes prone to experimental constraints that do not match the reality on the ground. 

In light of this, in the present study, we used a real-life clinical database containing rsEEG data of 102 patients at the SCI, MCI and AD stages. To our knowledge, this is the first study to date employing graph theory to characterize the evolution of brain networks throughout different clinical stages of cognitive decline, including healthy elders with subjective cognitive impairments (SCI), MCI patients and patients with AD. In the literature, many studies investigated network topology on different cognitive profiles [57,64,65,67,70,71]. Nevertheless, the majority of studies considered normal aged-matched healthy subjects as controls to study the evolution of EEG markers through MCI and AD stages. When SCI subjects were considered in [70], the authors studied different cognitive phenotype profiles in the population to predict the evolution towards dementia.

In addition, we performed a graph theory analysis based on functional connectivity values quantified with *EpEn*. This choice was made after comparing such a measure to two widely used metrics, the coherence and phase lag index, relying on different mathematical concepts. The experimental study was carried out in four frequency bands (delta, theta, alpha and beta) considering all the 30 rsEEG channels available in our data. 

The experiments showed that a statistical modeling of EEG with a spatiotemporal entropy measure (*EpEn*) allowed a better differentiation between the SCI, MCI and AD stages, compared to the coherence and phase lag index, which are deterministic measures. The average adjacency matrices of the three groups show a different connectivity organization with *EpEn* in the four frequency bands (Figure 4).

In delta, the AD group presented higher *EpEn* values compared to SCI group, especially in the prefrontal and frontal regions (Figure 4 and Figure 7). In alpha and beta, the opposite behavior appeared: the AD group showed lower *EpEn* values compared to the SCI group. *EpEn* measures the information content related to the dynamics of brain activity, conveyed by a couple of EEG time series. This finding shows that SCI subjects, considered as controls, presented a high information content in their rsEEG with brain dynamics between 8 and 30 Hz, while the AD group showed a slower rsEEG activity between 1 and 4 Hz. 

This result is in accordance with previously published studies: compared to the controls, AD patients showed an increase in slow rsEEG activities (delta and theta) and a decrease in fast rsEEG activities (alpha and beta) [9,19,20,21,22,23,24,25,79]. It is interesting to notice that these results are still valid although the control subjects in the present study are not considered as healthy subjects since they suffer from SCI with memory complaints with an increased risk of future objective cognitive decline. 

Regarding the MCI group, it exhibited the highest *EpEn* values in delta and the lowest *EpEn* values in alpha and beta (Figure 4 and Figure 7). These results show the slowing of rsEEG activity (low information content) in both MCI and AD relatively to SCI, since the activity is concentrated in the low frequency range, between 1 and 4 Hz. However, the MCI group displayed a stronger activity compared to AD (Figure 4 and Figure 7), which may reveal a compensatory mechanism underpinning the cognitive activity of MCI. These findings with the *EpEn* measure showed that the MCI group was intermediate between SCI and AD, which fits with certain results of the literature reporting that MCI is considered to be the transitional stage between normal aging and AD [25,54,67,71].

Graph theory was then applied on *EpEn* functional connectivity matrices using three core and complementary topological parameters: the clustering coefficient, shortest path and modularity. In contrast to a majority of works in the literature, we adopted, in the present study, a weighted graph analysis based on fully connected matrices. This allows preservation of all the available information. Moreover, it has been shown that weighted graph analysis could provide a richer topological information compared with classical binary analysis [65,66,80].

Our experiments showed that the analysis of functional connectivity in terms of its topological organization in the brain network, and not only in terms of its values, allows a better understanding on the evolution of functional connectivity networks throughout SCI, MCI and AD.

The clustering coefficient (Figure 9) showed the same behavior as *EpEn* (Figure 7): the AD group presented higher values in delta and lower values in alpha and beta compared to the SCI group. However, we noticed that the clustering coefficient led to a better distinction between the three populations compared to *EpEn*, especially in the theta band. This result shows the improvement brought by a global network analysis in addition to a local functional connectivity estimation, which is already efficient in characterizing the three populations. 

Previous studies reported an increased [53,54,67], decreased [51,52,55,57,65] or unmodified [50] clustering coefficient in AD compared to the control group. In the present study, the clustering coefficient was instead increased in AD and even more in MCI compared with SCI in the delta band and at all electrode locations. The opposite was observed in alpha and beta, with a reduced clustering coefficient in AD and even more in MCI. In theta, we observed the same tendency as in alpha and beta, except in the prefrontal and some part of frontal region (Figure 10b). In alpha, for which a consensus appears in the literature, our study confirms that AD leads to a reduction of the clustering coefficient.

The topological network analysis with clustering coefficient was found to be frequency band-dependent; this could partially explain the divergent results in the state of the art in addition to the data characteristics and methodological differences. This finding leads to interpreting the parameter differently depending on the frequency band.

In delta, the results indicate the ability of the AD and MCI networks to form locally dense cliques (high clustering coefficient); however, the MCI group presents a predominance of high-strength intrinsic connectivity. This may reflect the compensatory response to the neuronal damage occurring early in the disease process. Nevertheless, typically, the literature reports that a high value of the clustering coefficient reflects the robustness of a network in case of cognitive impairments or damage. This phenomenon was observed in our study, particularly in alpha and beta for the SCI group, since their rsEEG activity is contained in such high frequencies. 

Regarding the shortest path, SCI group showed, by far, the highest values of the shortest path in delta, and no difference was observed between MCI and AD (Figure 11a). In alpha and beta, the MCI group exhibited the highest shortest path values, which decreased in AD and SCI (Figure 11b,c). The difference between the three populations in terms of the shortest path was more notable in alpha for almost all EEG channels.

The information transfer in the AD and MCI networks appeared to be more fluent compared to SCI in delta. This suggests the establishment of more short-term connections in SCI at very low frequencies. On the contrary, in high-frequency ranges, especially in alpha, the low values of the shortest path indicate rapid and high efficiency in information transmission across the SCI network, reflecting more long-term connections in SCI compared to MCI and AD. This is in accordance with some studies reporting that AD leads to a decrease of the path length [42,55].

In addition, in high frequencies (Figure 11b,c), the MCI group showed higher shortest path values compared with AD, meaning that the information is processed throughout more short-term connections in MCI. This interesting result could also reflect the compensation mechanism in MCI: it could be thus postulated that MCI patients may exploit additional neural resources to compensate the loss of cognitive functions occurring early in the disease process.

This postulate is consistent with the high variance of local clustering coefficient values across electrodes, observed in MCI in alpha and beta (Figure 10c,d) and also observed with *EpEn* in Figure 7. In fact, when the homogeneity in the functional connectivity between nodes decreases, there is a high chance to transmit the information by means of several electrodes, leading to an increased shortest path.

Based on both segregation and integration parameters, we notice that, for high frequencies, the SCI group observed a simultaneously high clustering coefficient and low shortest path, meaning that SCI network tends to have a small world topology. Indeed, the small-world topology presents an optimal balance between local connected structure and global distributed information processing [44,46]. This occurs due to the existence of relatively few long-term connections, making the network more resistant to damage. Some studies have investigated the small-worldness of the brain network in AD context; they found that the AD group exhibits a more random overall network structure [50,51,52] compared with HC, which corresponds to our results for high frequencies.

Finally, when investigating the modular structure of the retrieved networks, we did not find differences between the three populations in terms of the modularity value. This may be due to the use of fully connected matrices. The majority of studies in the literature reported increased modularity in HC compared to AD, which indicates that the brain dynamics is organized into sub-autonomous networks that interact with one another through relatively short and long-term pathways. This brain structure is more resilient to neuronal damage. 

Nevertheless, when investigating the composition of the obtained modules, we found a difference between the three populations in alpha and beta (Figure 13 and Figure 14). When comparing SCI and AD in terms of the composition of the sub-networks (modules), the results showed that, on the one hand, the electrodes of the same brain region were distributed over several modules in SCI; on the other hand, some obtained modules in SCI were extended from anterior to posterior brain regions. This result may indicate the strong interaction between different brain regions for the SCI group compared to AD, hence, facilitating the information transfer and process. These findings show that, in the case of damage, the network is more resilient in SCI compared with MCI and even more compared with AD. This result corresponds to our conclusions with the clustering coefficient and the shortest path. 

Furthermore, the spatial distribution of the obtained modules for MCI was found to be intermediate between that of SCI and AD. This result confirms our previous finding: MCI is a transitional stage between normal aging and the dementia stage. 

## 5. Study Limitations

Our study presents certain limitations. The obtained results should be considered limited to the context of resting-state and scalp-level EEG connectivity analysis. It is largely acknowledged that sensor-level analysis is prone to the effects of volume conduction and poor signal-to-noise ratio. Currently, there is no method that guarantees discarding volume conduction effects [81]. One way to manage this issue consists in using connectivity measures that are relatively insensitive to these effects. In our study, we used different connectivity metrics, in particular the PLI measure, which is relatively insensitive to this effect, since it discards the zero lag component of the interaction [9,34,35,36]. 

In addition, the *EpEn* measure exploits HMM, whose structure is adapted for modeling neural dynamics underlying the observed EEG signal. In fact, HMM is a probabilistic model used to describe the evolution of observable events or signal realizations, which depend on internal factors that are not directly observed, called “hidden states”. The statistical modeling of multidimensional EEG signals allows obtaining a functional connectivity measure that is more robust to noise as demonstrated in [73]. In addition, the non-linear interaction between pairwise signals is modeled by a mixture of Gaussians at the level of an epoch, to address the problem of zero lag correlations. 

However, further investigation should be performed using the EEG source estimation approach, which is emerging as a potential method that addresses the effects of volume conduction [68,69,70,71,81]. In our study, the use of low-density EEG recordings did not allow performing a correct source connectivity analysis. Actually, there is evidence that increasing the number of electrodes provides greater accuracy in source estimation. Many studies exploited at least 64 electrodes to obtain satisfactory results [68,69,70,71] as also reported in [57]. 

The main aim of our study was to investigate differences between different clinical stages, using EEG data acquired on elderly and impaired patients in real-life clinical conditions. In such a context, it is difficult to use EEG recordings with high-density electrodes for cost, practical and comfort reasons. In another study, it would be of great interest to assess the effectiveness of *EpEn* considering scalp and source-reconstructed EEG networks on highly dense EEG data and to compare with the outcomes of the present work.

In addition, our results are based on weighted graphs in which all nodes are connected. The majority of works in the literature performed a graph analysis after applying a thresholding to maintain only the strongest connections. It is thus important in the future to confront our study to different topological scales and analyze the topological organization between the three groups at different resolution scales. This is of high interest since topological parameters and group contrasts may differ across thresholds [65,71,82].

Furthermore, the present study analyzed the three populations by averaging across subjects to retrieve a general trend on the evolution towards dementia. An additional work should be conducted to investigate the brain network topology at the individual level, in order to gain a better understanding on the variability of the topology inside the same population.

Finally, the presented results were reported using a clinical database that includes 102 subjects acquired in real-life conditions. However, our findings need to be validated on other data in order to be confirmed. This study is therefore a preliminary work that requires conducting future in-depth research, which should involve more patients. One of the objectives of this forthcoming research will also be to go further in our analysis by comparing our EEG-based results with available neuropsychological and clinical markers.

## 6. Conclusions

The present study on rsEEG investigated brain network analysis over different stages of cognitive decline from SCI to AD passing through MCI. We proposed a new framework to study the topological brain networks based on a refined spatiotemporal entropy measure (*EpEn*), relying on a statistical modeling of EEG time series using HMM. This modeling approach is suitable to the analysis of the underlying neuronal dynamics, since it quantifies piecewise stationary epochs, the information content conveyed by EEG signals locally over time and spatially by estimating inter-channel relationships.

Our results add evidence for the comprehension of the progression of cognitive severity towards dementia. Our experiments demonstrated that functional connectivity and graph analysis was frequency band-dependent, and functional alterations started at the MCI stage with a specific scheme. In delta, the SCI group exhibited a reduction of brain activity quantified by *EpEn*, a decrease of clustering coefficient and an increase of the path length compared to MCI and AD. 

This indicates the ability of AD and MCI networks to form locally dense cliques. In high frequencies, especially in alpha, the opposite behavior appeared, suggesting a rapid and high efficiency in information transmission across the SCI network. We concluded that the brain network at SCI stage tends to have a small world topology compared to MCI and AD stages. Moreover, the modular structure of brain networks has revealed that, in the case of damage, the SCI network is more resilient to neuronal damage compared to that of MCI and even more compared to that of the AD stage.

Finally, our results add new pieces of evidence in the understanding of early brain changes, confirming that MCI is a transitional stage between SCI and AD. In addition, all the results pointed to the predominance of high-strength intrinsic connectivity that appears at the MCI stage, which may reflect the compensatory response to the neuronal damage occurring early in the disease process.

## Figures and Tables

**Table 1:** Clinical characteristics of the cohort.

Characteristics	SCI (*n* = 22)	MCI (*n* = 52)	AD (*n* = 28)
Age (mean ± SD)	68.9 ± 10.3	75.2 ± 10.8	80.8 ± 10.5
Female (%)	81.8%	61.5%	67.8%
MMSE (mean ± SD)	28.3 ± 1.6	24.5±4.9	18.3 ± 6.1
Benzodiazepine use (%)	4 (18.2%)	5 (9.6%)	8 (28.6%)
Antidepressant use (%)	2 (9%)	10 (19.2%)	12 (42.8%)
Neuroleptic use (%)	0	2 (3.8%)	5 (17.8%)
Hypnotic use (%)	5 (22.7%)	12 (23.1%)	7 (25%)

## Data Availability

We conducted a retrospective study on EEG data of patients referred to the outpatient memory clinic of the Charles-Foix hospital (France). This retrospective study was approved by the institutional review board of the local Ethics Committee Paris 6, on 16 May 2013 (France). All data were fully anonymized before exploiting them in our research work. Before conducting this retrospective study, an information letter on the research study was sent to the patients, with the possibility of them opposing the use of their collected data. Informed consent was thus waived in this context. Data are available upon request to researchers qualified to handle confidential data. Data requests can be made to Laurent Capelle (email: cppidf6.salpetriere@yahoo.fr, Comité de protection des personnes Ile-de-France VI) or from the corresponding author (kiyoka.kinugawa@aphp.fr) upon reasonable request. There are legal and ethical restrictions on sharing these data. The French law requires patients to be duly informed of any use of their data. We did not obtain patients’ permission to share their data publicly. We are not able to ask for their consent today because the data was collected some time ago, between 2009 and 2013.

## References

[1] Querfurth H.W., La Ferla F.M. (2010). Alzheimer’s disease. N. Engl. J. Med..

[2] Forner S., Baglietto-Vargas D., Martini A.C., Trujillo-Estrada L., La Ferla F.M. (2017). Synaptic Impairment in Alzheimer’s Disease: A Dysregulated Symphony. Trends Neurosci..

[3] Prince M.J., Wimo A., Guerchet M.M., Ali G.C., Wu Y.-T., Prina M. (2015). World Alzheimer Report 2015—The Global Impact of Dementia: An Analysis of Prevalence, Incidence, Cost and Trends.

[4] Jessen F., Amariglio R.E., Van Boxtel M., Breteler M., Ceccaldi M., Chételat G., Dubois B., Dufouil C., Ellis K.A., van der Flier W.M. (2014). A conceptual framework for research on subjective cognitive decline in preclinical Alzheimer’s disease. Alzheimer’s Dement..

[5] Mitchell A.J., Beaumont H., Ferguson D., Yadegarfar M., Stubbs B. (2014). Risk of dementia and mild cognitive impairment in older people with subjective memory complaints: Meta-analysis. Acta Psychiatr. Scand..

[6] Dubois B., Hampel H., Feldman H.H., Scheltens P., Aisen P., Andrieu S., Bakardjian H., Benali H., Bertram L., Blennow K. (2016). Preclinical Alzheimer’s disease: Definition; natural history; and diagnostic criteria. Alzheimer’s Dement..

[7] Petersen R.C., Roberts R.O., Knopman D.S., Boeve B.F., Geda Y.E., Ivnik R.J., Smith G.E., Jack C.R. (2009). Mild cognitive impairment: Ten years later. Arch. Neurol..

[8] Weiner M.W., Veitch D.P., Aisen P.S., Beckett L.A., Cairns N.J., Green R.C., Harvey D., Jack C.R., Jagust W., Liu E. (2013). The Alzheimer’s disease neuroimaging initiative: A review of papers published since its inception. Alzheimer’s Dement..

[9] Babiloni C., Blinowska K., Bonanni L., Cichocki A., De Haan W., Del Percio C., Dubois B., Escudero J., Fernández A., Frisoni G. (2020). What electrophysiology tells us about Alzheimer’s disease: A window into the synchronization and connectivity of brain neurons. Neurobiol. Aging.

[10] Zhang Y., Wang C., Sun C., Zhang X., Wang Y., Qi H., Ming D. (2015). Neural complexity in patients with poststroke depression: A resting EEG study. J. Affect. Disord..

[11] Lei Y., Belkacem A.N., Wang X., Sha S., Wang C., Chen C. (2022). A convolutional neural network-based diagnostic method using resting-state electroencephalograph signals for major depressive and bipolar disorders. Biomed. Signal Processing Control.

[12] Rachamanee S., Wongupparaj P. (2021). Resting-state EEG datasets of adolescents with mild, minimal, and moderate depression. BMC Res. Notes.

[13] Prasanna J., Subathra M.S.P., Mohammed M.A., Damaševičius R., Sairamya N.J., George S.T. (2021). Automated Epileptic Seizure Detection in Pediatric Subjects of CHB-MIT EEG Database—A Survey. J. Pers. Med..

[14] Xu P., Xiong X., Xue Q., Li P., Zhang R., Wang Z., Valdes-Sosa P.A., Wang Y., Yao D. (2014). Differentiating between psychogenic nonepileptic seizures and epilepsy based on common spatial pattern of weighted EEG resting networks. IEEE Trans. Biomed. Eng..

[15] Faiman I., Smith S., Hodsoll J., Young A.H., Shotbolt P. (2021). Resting-state EEG for the diagnosis of idiopathic epilepsy and psychogenic nonepileptic seizures: A systematic review. Epilepsy Behav..

[16] Yi G.S., Wang J., Deng B., Wei X.L. (2017). Complexity of resting-state EEG activity in the patients with early-stage Parkinson’s disease. Cogn. Neurodynamics.

[17] Emamzadeh-Hashemi E.A., Mahdizadeh A., Mirian M.S., Lee S., McKeown M.J. (2022). Deep Transfer Learning for Parkinson’s Disease Monitoring by Image-Based Representation of Resting-State EEG Using Directional Connectivity. Algorithms.

[18] Fonseca L.C., Tedrus G.M.A.S., Carvas P.N., Machado E.C.F.A. (2013). Comparison of quantitative EEG between patients with Alzheimer’s disease and those with Parkinson’s disease dementia. Clin. Neurophysiol..

[19] Babiloni C., Lizio R., Marzano N., Capotosto P., Soricelli A., Triggiani A.I., Susanna C., Loreto G., Del Claudio P. (2016). Brain neural synchronization and functional coupling in Alzheimer’s disease as revealed by resting state EEG rhythms. Int. J. Psychophysiol..

[20] Jelic V., Johansson S.E., Almkvist O., Shigeta M., Julin P., Nordberg A., Winblad B., Wahlund L.-O. (2000). Quantitative electroencephalography in mild cognitive impairment: Longitudinal changes and possible prediction of Alzheimer’s disease. Neurobiol. Aging.

[21] Brassen S., Adler G. (2003). Short-term effects of acetylcholinesterase inhibitor treatment on EEG and memory performance in Alzheimer patients: An open; controlled trial. Pharmacopsychiatry.

[22] Onofrj M., Thomas A., Iacono D., Luciano A.L., Di Iorio A. (2003). The effects of a cholinesterase inhibitor are prominent in patients with fluctuating cognition: A part 3 study of the main mechanism of cholinesterase inhibitors in dementia. Clin. Neuropharmacol..

[23] Ponomareva N.V., Selesneva N.D., Jarikov G.A. (2003). EEG alterations in subjects at high familial risk for Alzheimer’s disease. Neuropsychobiology.

[24] Jeong J. (2004). EEG dynamics in patients with Alzheimer’s disease. Clin. Neurophysiol..

[25] Dauwels J., Vialatte F., Cichocki A. (2010). Diagnosis of Alzheimer’s disease from EEG signals: Where are we standing?. Curr. Alzheimer Res..

[26] Adeli H., Ghosh-Dastidar S., Dadmehr N. (2008). A spatio-temporal wavelet-chaos methodology for eeg based diagnosis of Alzheimer’s disease. Neurosci. Lett..

[27] Lake D.E., Richman J.S., Griffin M.P., Moorman J.R. (2002). Sample entropy analysis of neonatal heart rate variability. Am. J. Physiol. Regul. Integr. Comp. Physiol..

[28] Abasolo D., Hornero R., Espino P., Alvarez D., Poza J. (2006). Entropy analysis of the EEG background activity in Alzheimer’s disease patients. Physiol. Meas..

[29] De Bock T., Das S., Mohsin M., Munro N.B., Hively L.M., Jiang Y., Smith C.D., Wekstein D.R., Jicha G.A., Lawson A. Early detection of Alzheimer’s disease using nonlinear analysis of EEG via Tsallis entropy. Proceedings of the 2010 Biomedical Sciences and Engineering Conference.

[30] Abasolo D., Hornero R., Espino P., Poza J., Sanchez C.I., de la Rosa R. (2005). Analysis of regularity in the EEG background activity of Alzheimer’s disease patients with approximate entropy. Clin. Neurophysiol..

[31] Pincus S.M. (2006). Approximate entropy as a measure of irregularity for psychiatric serial metrics. Bipolar Disord..

[32] Escudero J., Basolo D., Hornero R., Espino P., Lopez M. (2006). Analysis of electroencephalograms in Alzheimer’s disease patients with multiscale entropy. Physiol. Meas..

[33] Dauwels J., Vialatte F.B., Musha T., Cichocki A. (2010). A comparative study of synchrony measures for the early diagnosis of Alzheimer’s disease based on EEG. Neuroimage.

[34] Bastos A.M., Schoffelen J.M. (2015). A Tutorial Review of Functional Connectivity Analysis Methods and Their Interpretational Pitfalls. Front. Syst. Neurosci..

[35] Kasakawa S., Yamanishi T., Takahashi T., Ueno K., Kikuchi M., Nishimura H., Chen Y.W., Torro C., Tanaka S., Howlett R.C., Jain L. (2016). Approaches of Phase Lag Index to EEG Signals in Alzheimer’s Disease from Complex Network Analysis. Innovation in Medicine and Healthcare 2015.

[36] Stam C.J., Nolte G., Daffertshofer A. (2007). Phase lag index: Assessment of functional connectivity from multi channel EEG and MEG with diminished bias from common sources. Hum. Brain Mapp..

[37] Sankari Z., Adeli H., Adeli A. (2012). Wavelet coherence model for diagnosis of Alzheimer’s disease. Clin. EEG Neurosci..

[38] Adler G., Brassen S., Jajcevic A. (2003). EEG coherence in Alzheimer’s dementia. J. Neural Transm..

[39] Locatelli T., Cursi M., Liberati D., Franceschi M., Comi G. (1998). EEG coherence in Alzheimer’s disease. Electroencephalogr. Clin. Neurophysiol..

[40] Besthorn C., Förstl H., Geiger-Kabisch C., Sattel H., Gasser T., Schreiter-Gasser U. (1994). EEG coherence in Alzheimer disease. Electroencephalogr. Clin. Neurophysiol..

[41] Jeong J., Gore J., Peterson B. (2001). Mutual information analysis of the EEG in patients with Alzheimer’s disease. Clin. Neurophysiol..

[42] Czigler B., Csikós D., Hidasi Z., Anna Gaál Z., Csibri E., Kiss E., Salacz P., Molnár M. (2008). Quantitative EEG in early Alzheimer’s disease patients-power spectrum and complexity features. Int. J. Psychophysiol..

[43] Pijnenburg Y.A., V d Made Y., Van Cappellen van Walsum A.M., Knol D.L., Scheltens P., Stam C.J. (2004). EEG Synchronization likelihood in MCI and AD during a working memory task. Clin. Neurophysiol..

[44] Bullmore E., Sporns O. (2009). Complex brain networks: Graph theoretical analysis of structural and functional systems. Nat. Rev. Neurosci..

[45] Bassett D.S., Bullmore E.T. (2009). Human brain networks in health and disease. Curr. Opin. Neurol..

[46] Rubinov M., Sporns O. (2010). Complex network measures of brain connectivity: Uses and interpretations. Neuroimage.

[47] Stam C.J., van Straaten E.C.W. (2012). The organization of physiological brain networks. Clin. Neurophysiol..

[48] Stam C.J. (2014). Modern network science of neurological disorders. Nat. Rev. Neurosci..

[49] Sporns O. (2018). Graph theory methods: Applications in brain networks. Dialogues Clin. Neurosci..

[50] Stam C.J., Jones B.F., Nolte G., Breakspear M., Scheltens P. (2007). Small-world networks and functional connectivity in Alzheimer’s disease. Cereb. Cortex.

[51] Stam C.J., de Haan W., Daffertshofer A., Jones B.F., Manshanden I., van Cappellen van Walsum A.M., Montez T., Verbunt J.P.A., de Munck J.C., van Dijk B.W. (2009). Graph theoretical analysis of magnetoencephalographic functional connectivity in Alzheimer’s disease. Brain.

[52] de Haan W., Pijnenburg Y.A., Strijers R.L., van der Made Y., van der Flier W.M., Scheltens P., Stam C.J. (2009). Functional neural network analysis in frontotemporal dementia and Alzheimer’s disease using EEG and graph theory. BMC Neurosci..

[53] He Y., Chen Z., Evans A. (2008). Structural insights into aberrant topological patterns of large-scale cortical networks in Alzheimer’s disease. J. Neurosci..

[54] Yao Z., Zhang Y., Lin L., Zhou Y., Xu C., Jiang T. (2010). Abnormal cortical networks in mild cognitive impairment and Alzheimer’s disease. PLoS Comput. Biol..

[55] Tijms B.M., Wink A.M., de Haan W., van der Flier W.M., Stam C.J., Scheltens P., Barkhof F. (2013). Alzheimer’s disease: Connecting findings from graph theoretical studies of brain networks. Neurobiol. Aging.

[56] Miraglia F., Vecchio F., Bramanti P., Rossini P.M. (2016). EEG characteristics in “eyes-open” versus “eyes-closed” conditions: Smallworld network architecture in healthy aging and age-related brain degeneration. Clin. Neurophysiol..

[57] Franciotti R., Falasca N.W., Arnaldi D., Famà F., Babiloni C., Onofrj M., Nobili F.M., Bonanni L. (2019). Cortical Network Topology in Prodromal and Mild Dementia Due to Alzheimer’s Disease: Graph Theory Applied to Resting State EEG. Brain Topogr..

[58] He Y., Chen Z., Gong G., Evans A. (2009). Neuronal networks in Alzheimer’s disease. Neuroscientist.

[59] Wang J., Yang C., Wang R., Yu H., Cao Y., Liu J. (2016). Functional brain networks in Alzheimer’s disease: EEG analysis based on limited penetrable visibility graph and phase space method. Physical A.

[60] Afshari S., Jalili M. (2017). Directed functional networks in Alzheimer’s disease: Disruption of global and local connectivity measures. IEEE J. Biomed. Health Inform..

[61] McBride J., Zhao X., Munro N., Smith C., Jicha G., Jiang Y. (2013). Resting EEG discrimination of early stage Alzheimer’s disease from normal aging using inter-channel coherence network graphs. Ann. Biomed. Eng..

[62] Jalili M. (2017). Graph theoretical analysis of Alzheimer’s disease: Discrimination of AD patients from healthy subjects. Inf. Sci..

[63] Miraglia F., Vecchio F., Rossini P.M. (2017). Searching for signs of aging and dementia in EEG through network analysis. Behav. Brain Res..

[64] Das S., Puthankattil S.D. (2020). Complex network analysis of MCI-AD EEG signals under cognitive and resting state. Brain Res..

[65] Jiangkuan C., Liu C., Peng C.K., Fuh J.L., Hou F., Yang A.C. (2019). Topological reorganization of EEG functional network is associated with the severity and cognitive impairment in Alzheimer’s disease. Phys. A Stat. Mech. Its Appl..

[66] Mehraram R., Kaiser M., Cromarty R., Graziadio S., O’Brien J., Killen A., Taylor J.P., Peraza L.R. (2019). Weighted network measures reveal differences between dementia types: An EEG study. Hum. Brain Mapp..

[67] Vecchio F., Miraglia F., Marra C., Quaranta D., Vita M.G., Bramanti P., Rossini P.M. (2014). Human brain networks in cognitive decline: A graph theoretical analysis of cortical connectivity from EEG data. J. Alzheimer’s Dis..

[68] Hassan M., Wendling F. (2018). Electroencephalography source connectivity: Toward high time/space resolution brain networks. arXiv.

[69] Tait L., Stothart G., Coulthard E., Brown J.T., Kazanina N., Goodfellow M. (2019). Network substrates of cognitive impairment in Alzheimer’s Disease. Clin. Neurophysiol..

[70] Gaubert S., Raimondo F., Houot M., Corsi M.-C., Sitt J.D., Hermann B., Oudiette D., Gagliardi G., Habert M.-O. (2019). EEG evidence of compensatory mechanisms in preclinical Alzheimer’s disease. Brain.

[71] Dattola S., Mammone N., Morabito F.C., Rosaci D., Sarné G.M.L., La Foresta F. (2021). Testing Graph Robustness Indexes for EEG Analysis in Alzheimer’s Disease Diagnosis. Electronics.

[72] Houmani N., Vialatte F.B., Latchoumane C., Jeong J., Dreyfus G. Stationary Epoch-based Entropy Estimation for Early Diagnosis of Alzheimer’s Disease. Proceedings of the 12th Low Voltage Low Power Conference, IEEE FTFC 2013.

[73] Houmani N., Vialatte F.B., Dreyfus G. (2015). Epoch-based Entropy for Early Screening of Alzheimer’s Disease. Int. J. Neural Syst..

[74] Houmani N., Vialatte F.B., Gallego-Jutglà E., Dreyfus G., Nguyen-Michel V.-H., Mariani J., Kinugawa K. (2018). Diagnosis of Alzheimer’s disease with Electroencephalography in a differential framework. PLoS ONE.

[75] Houmani N., Abazid M., De Santiago K., Boudy J., Dorizzi B., Mariani J., Kinugawa-Bourron K., Yurish S. (2021). EEG signal analysis with a statistical entropy-based measure for Alzheimer’s disease detection, open access book. Advances in Signal Processing: Reviews, Book Series.

[76] Abazid M., Houmani N., Boudy J., Dorizzi B., Mariani J., Kinugawa-Bourron K. (2021). A comparative study of functional connectivity measures for brain network analysis in the context of AD detection with EEG. Entropy.

[77] McKeith I.G., Dickson D.W., Lowe J., Emre M., O’Brien J.T., Feldman H., Cummings J., Duda J.E., Lippa C., Perry E.K. (2005). Diagnosis and management of dementia with Lewy bodies. Third report of the DLB consortium. Neurology.

[78] Newman M.E.J., Girvan M. (2004). Finding and evaluating community structure in networks. Phys. Rev. E.

[79] Bennys K., Rondouin G., Vergnes C., Touchon J. (2001). Diagnostic value of quantitative EEG in Alzheimer’s disease. Clin. Neurophysiol..

[80] Rubinov M., Sporns O. (2011). Weight-conserving characterization of complex functional brain networks. Neuroimage.

[81] Lai M., Demuru M., Hillebrand A., Fraschini M. (2018). A comparison between scalp- and source-reconstructed EEG networks. Sci. Rep..

[82] Garrison K.A., Scheinost D., Finn E.S., Shen X., Constable R.T. (2015). The (in)stability of functional brain network measures across thresholds. NeuroImage.

